# Deploying an NFV-Based Experimentation Scenario for 5G Solutions in Underserved Areas

**DOI:** 10.3390/s21051897

**Published:** 2021-03-08

**Authors:** Victor Sanchez-Aguero, Ivan Vidal, Francisco Valera, Borja Nogales, Luciano Leonel Mendes, Wheberth Damascena Dias, Alexandre Carvalho Ferreira

**Affiliations:** 1 IMDEA Networks Institute, Avda. del Mar Mediterráneo, 22, 28918 Madrid, Spain; 2 Department of Telematic Engineering, Universidad Carlos III de Madrid, 28911 Leganes, Spain; ividal@it.uc3m.es (I.V.); fvalera@it.uc3m.es (F.V.); bdorado@pa.uc3m.es (B.N.); 3 Instituto Nacional de Telecomunicações, Santa Rita do Sapucaí 37540-000, Brazil; lucianol@inatel.br (L.L.M.); wheberth@gmail.com (W.D.D.); alexandrecf@inatel.br (A.C.F.)

**Keywords:** 5th generation cellular networks (5G), remote area network, Small Unmanned Aerial Vehicles (SUAVs), Network Functions Virtualization (NFV), 3rd Generation Partnership Project (3GPP)

## Abstract

Presently, a significant part of the world population does not have Internet access. The fifth-generation cellular network technology evolution (5G) is focused on reducing latency, increasing the available bandwidth, and enhancing network performance. However, researchers and companies have not invested enough effort into the deployment of the Internet in remote/rural/undeveloped areas for different techno-economic reasons. This article presents the result of a collaboration between Brazil and the European Union, introducing the steps designed to create a fully operational experimentation scenario with the main purpose of integrating the different achievements of the H2020 5G-RANGE project so that they can be trialed together into a 5G networking use case. The scenario encompasses (i) a novel radio access network that targets a bandwidth of 100 Mb/s in a cell radius of 50 km, and (ii) a network of Small Unmanned Aerial Vehicles (SUAV). This set of SUAVs is NFV-enabled, on top of which Virtual Network Functions (VNF) can be automatically deployed to support occasional network communications beyond the boundaries of the 5G-RANGE radio cells. The whole deployment implies the use of a virtual private overlay network enabling the preliminary validation of the scenario components from their respective remote locations, and simplifying their subsequent integration into a single local demonstrator, the configuration of the required GRE/IPSec tunnels, the integration of the new 5G-RANGE physical, MAC and network layer components and the overall validation with voice and data services.

## 1. Introduction and Motivation

The fifth generation of cellular network technology (5G) is becoming a reality. Researchers and industry are working conscientiously to build ground-breaking solutions that increase transmission speeds, reduce latency, decrease energy consumption, or improve connectivity. The 3rd Generation Partnership Project (3GPP) [1] is working on a series of releases to compose the 5G network scenarios that are being developed to fulfil the requirements imposed by enhanced Mobile Broadband (eMBB) [2], Ultra-Reliable Low Latency Communications (URLLC) [3], and massive Machine Type Communications (mMTC) [4]. Release 15 [5] of 3GPP specifications focused on high data rates for eMBB, with a peak downlink rate of 20 Gb/s and 10 Gb/s uplink rate. Release 16 [6] is focusing on reducing the end-to-end latency and increasing robustness for URLLC, and Release 17 [7] is under specification to reduce power consumption on power-limited devices and increase the number of connections for mMTC.

Besides these promising improvements, presently the International Telecommunication Union estimates that 46.4% of the world population cannot properly access the Internet, and connectivity in remote/low populated areas is normally considered to require considerable effort and investments [8]. However, one of the key performance indicators [9] to be achieved at an operational level of the 5G ecosystem, is to provide ubiquitous access including rural and remote areas. In continental-size countries, such as Brazil, one major application scenario is the remote and rural area coverage, where a large parcel of the population is living without connectivity and where informatization of the fields is required to increase farms productivity. Since costs are the major hurdle for mobile network operators, 5G must also present an operation mode that allows for extensive coverage, reducing the number of base stations and spectrum costs. In addition, this solution will have substantial social benefits, such as guaranteeing universal access to education and healthcare, or the digital integration of different isolated communities and countries.

Over the past few years, there has been considerable proliferation of experimentation projects in 5G technologies. In general, there is no commercial deployment of the 5G technology (most of the deployments are still based on legacy infrastructure and standalone 5G solutions have not been deployed yet in many places). Accordingly, most of the experimentation is done under consortium projects involving relevant entities, including end users, technology developers, and operators. In Europe, many projects have been funded by the European Union to provide 5G experimentation facilities and foster the adoption of 5G technologies by vertical sectors. These projects include 5G-VINNI [10], the 5GINFIRE [11], the 5Growth [12], the 5G-DIVE [13], or the 5G-EVE [14].

**Figure 1:**
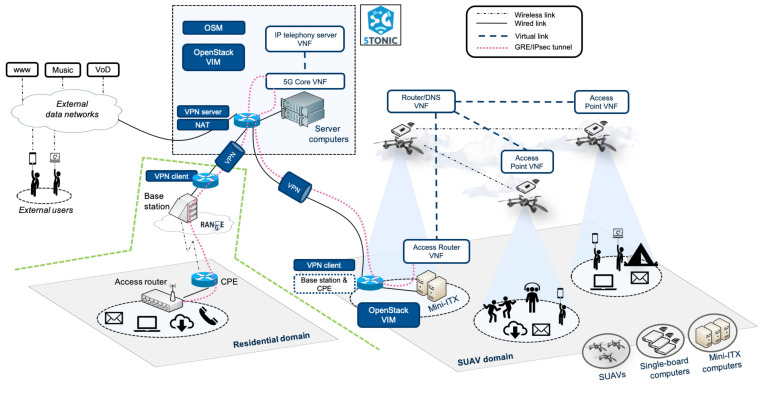
Overview of the testbed components and the experimentation scenario.

This article presents an experience report after building a use case scenario with 5G technologies (Figure 1) within the Horizon 2020 5G-RANGE (Remote area Access Network for 5th GEneration) project [15], a 3-year European and Brazilian cooperation effort, whose main objective is the design and implementation of a remote area access network solution under 3GPP specifications and the 5G standard technologies. The contribution presented in this paper has been deployed at the Instituto Nacional de Telecomunicações (Inatel), in Santa Rita do Sapucaí, Brazil, and the 5G Telefonica Open Network Innovation Centre laboratory (5TONIC) [16] in Madrid, Spain. 5TONIC is one of the leading laboratories for 5G experimentation based in Madrid, Spain. The 5TONIC laboratory is established to provide an open ecosystem where members from business, industry, and academia collaborate with the telecoms research projects.

Previous works by the authors present two of the main achievements of 5G-RANGE such as a multi-site Network Function Virtualization (NFV) testbed designed for experimentation with Small Unmanned Aerial Vehicles (SUAV) [17], or the new physical (radio) and MAC (Medium Access Control) layers developed to overcome the long-range link under 5G key performance indicators, targeting a cell radius of 50 Km with 100 Mb/s at the edge and closing the connectivity gap in remote and rural areas [18].

However, this article shows the whole experimentation scenario that has been built integrating both solutions and presents them in two different domains that can interact with each other. One of the domains includes the physical and MAC layers that allow for Dynamic Spectrum Allocation [19] by exploiting vacant TV channels using Cognitive Radio techniques [20]. Spectrum sensing, low out-of-band emissions, fragmented spectrum access [21] and cognitive cycle are some of the features that must be incorporated in the physical and MAC layers to allow 5G networks to be used to close the connectivity gap in remote and rural areas. The other domain includes the SUAV scenario where network functions can be automatically deployed to support casual network communications beyond the boundaries of the 5G-RANGE radio cells, or to cover shadow areas within the radio cells themselves (e.g., fire extinction, search and rescue operations, festive events, field inspections).

The article is also showing the methodology to perform this type of complex integrations with different domains located in remote locations. The domains are connected using a VPN-based overlay network (so that they can transparently be deployed anywhere) and through a baseline 5G core software.

In addition, it is shown how all the network layer components in both domains (as suggested by the 3GPP) are virtualized and executed as Virtualized Network Functions (VNFs), using a Management and Orchestration (MANO) platform defined by the ETSI in the context of NFV paradigm. Wireless APs that offer network access connectivity to end users within their vicinity may be deployed as VNFs in several SUAVs, some other SUAVs or Ground Units could deploy virtualized network routers, supporting the exchange of data between users in the geographic area, or a gateway function can be available at specific locations, enabling for data communications between users and external data networks through a 5G Core (which would also be provided in the form of one or several VNFs in the operator domain). The article shows how this approach enables an easier and more flexible incorporation of additional domains to the scenario, aiding the development of proof-of-concept activities involving Internet, third-party, and operator-specific services, such as web browsing, email, video on demand, and IP telephony. Finally, the article provides different measurements performed over the infrastructure.

## 2. Background on 5G-RANGE Technologies

To support effective network communications over remote areas, 5G-RANGE has followed a practical approach: (i) the development of novel physical and MAC layer mechanisms, able to efficiently handle data communications over long distances; (ii) the adoption of well-known and widely used technologies, as well as recognized standards under development in the context of 5G networking, to provide end-user terminals with end-to-end network connectivity, and support the provision of an operator, third-party, and Internet services. In addition, the design of mechanisms to enable cost-effective network services over delimited geographic areas (e.g., to support network communications beyond the boundaries of the 5G-RANGE radio cells) is also considered.

This section identifies and provides a brief overview of these technologies, outlining the technological background that has driven the design and the development of the experimental testbed described in the following section.

### 2.1. Physical and MAC Layers

The 5G-RANGE project has developed an innovative new radio physical layer using a powerful channel code scheme to enable a robust long-range link. A polar code [22] with a variable coding rate is used to protect the data received from the upper layers, increasing the system performance in terms of bit and block error rates. The encoded data are mapped into Quadrature Amplitude Modulation (QAM) symbols, where modulation order can be selected according to the channel conditions (from 4-QAM up to 256-QAM).

After the QAM mapping, the data symbols are applied to the Multiple-Input Multiple-Output (MIMO) block, which can operate in two different modes. In the first mode, a space-time block coding [23] is used to enhance the system robustness for users located far from the base station. This mode allows the receiver to combine the data transmitted by the two antennas, resulting in a maximum diversity gain that is twice the number of receive antennas. The second operation relies on space multiplexing [24], where the two transmit antennas are used to send different data blocks, doubling the overall data rate of the system. Typically, space-time block coding is used to enhance the system robustness for users located far from the base station, while space multiplexing is used to increase the data rate for those users close to it.

This physical layer is controlled and configured by the MAC layer, according to the channel conditions. The main novelty introduced at this layer is the cognitive cycle, exploiting vacant TV channels using cognitive radio techniques [20]. The User Equipment (UE) can be instructed by the base station to perform a spectrum measurement at its location infers whether the channel is available or occupied. The Collaborative Spectrum Sensing Optimized for Remote Areas [25] block acquires samples collected from the channel and each requested UE performs a Primary User detection algorithm. The outcome of this measurement is one of two hypotheses: H0, stating that the channel is available or H1, stating that the channel is occupied. The measurements are sent to the base station, where a dynamic spectrum allocation [19] function is responsible to fuse all the measurements into a single decision variable. Data from a geolocation database can also be used to aid the decision process, i.e., allowing the selection of the channels that shall be investigated as potential idle channels. Once a set of channels is identified as vacant, a resource scheduler allocates the user data to protocol data units, which will be delivered to the physical layer. These protocol data units also carry the configuration of the physical layer to be employed by each user (e.g., code rate, QAM mapper and MIMO scheme). Data recovered by the physical layer are also delivered to the MAC layer using the same protocol data unit structure.

### 2.2. Network Layer

**Figure 2:**
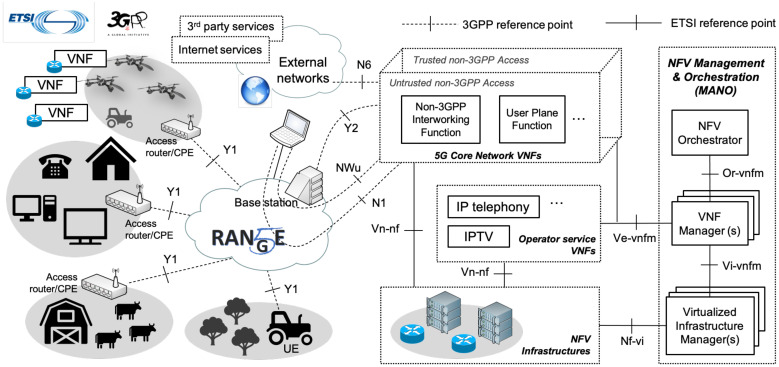
High-level overview of the 5G-RANGE architecture.

The network layer has a fundamental role to support end-user communications. It complements the physical and MAC layers of the radio access network with the required features to provide the UE with secure end-to-end network connectivity towards other UE and external networks. Figure 2 outlines the role of the network layer within the 5G-RANGE architecture.

The connectivity service offered by the network layer is realized with the use of a 5G core network, as defined by the 3GPP [1]. The 5G core network identifies a set of well-defined protocols and interfaces allowing the interconnection of non-3GPP access networks (e.g., the 5G-RANGE access network), which may support trusted or untrusted connectivity to a 5G core network or, more generally, to a public mobile network infrastructure. Moreover, it enables the establishment of protected communications towards the UE, using standard tunneling protocols over the access network (for non-3GPP accesses, these are GRE (Generic Routing Encapsulation) [26] and IPsec (Internet Protocol security) [27]).

From an architectural perspective, the 5G-RANGE network layer relies on ETSI Network Functions Virtualization (NFV). This way, the different elements of the 5G core network, as well as the constituent functions of operator and third-party services, can be executed as Virtual Network Functions (VNFs). The lifecycle management of all the VNFs framed at the network layer follows the procedures indicated by the ETSI Management and Orchestration (MANO) framework. Figure 2 outlines the 5G-RANGE architecture.

In 5G-RANGE, ETSI NFV is also considered to support the cost-effective deployment of lightweight network functions on localized remote geographic areas. In this respect, the approach taken by the project leverages resource-constrained platforms that can be embedded in vehicles that may exist or be deployed in the remote area (e.g., SUAVs, harvesters, tractors, etc.). These vehicles are consequently transformed into functional mobile compute nodes, offering computing, storage and network resources that can be under the control of a MANO platform to support the execution of VNFs. They have the potential to be placed around specific locations and be interconnected, allowing the on-demand creation of functional NFV infrastructures over localized geographic areas. This NFV infrastructure can be used to complement the access network resources, and provide cost-effective telecommunication services controlled by a MANO platform. This way, the proposed approach allows the on-demand creation of a functional NFV infrastructure over a localized geographic area. This infrastructure can be used to complement the access network resources and provision cost-effective telecommunication services under the control of a MANO platform. In addition, the NFV infrastructure facilitates the dissemination of data across the deployment area, through the multi-hop ad hoc network built by the mobile compute nodes.

In 5G-RANGE, this approach has been explored in different use cases, including service provisioning beyond the boundary of radio cells [28], supporting emergency communication services in remote areas [29], and disseminating vertical-specific data, particularly in smart farming scenarios [17].

### 2.3. NFV Testbed Description and VNF Repository

As previously commented, the goal of this work is to build the scenario shown in Figure 1. For this purpose, we have used an NFV testbed that is available at the 5TONIC laboratory [16] based in Madrid, Spain. A detailed description of the NFV testbed can be found in [17]. For the sake of completeness, in the following we present a summary of its main features and components. The testbed includes a functional MANO platform based on Open-Source MANO (OSM), an ETSI-hosted project providing an NFV orchestration software stack. A well-known and widely adopted cloud computing solution, OpenStack, was selected to provide the Virtual Infrastructure Manager (VIM) functionalities. Both the OSM stack and the OpenStack controller run in independent virtual machines, easing the management of the MANO components and their vertical scaling to satisfy operational needs. The MANO platform supports the automated deployment of VNFs over an NFV infrastructure composed by three server computers, accounting for a total of 24 vCPUs, 96 GB of RAM, and 6 TB of storage. These VNFs may also access Internet services, through a Network Address Translation (NAT) function provided at 5TONIC edge router.

To increase the potential for experimentation with 5G-RANGE technologies, the baseline testbed supports the flexible incorporation of network domains, by means of an overlay network architecture based on a Virtual Private Network (VPN) service hosted at 5TONIC. Each of these domains may include hardware and software components prototyped by 5G-RANGE partners. It may also host an NFV infrastructure that can be attached to the 5TONIC MANO platform. This way, the addition of new domains allows configuring moderately complex experimentation scenarios, enabling the validation of 5G-RANGE developments along with other assets produced or adopted by the project. Each network domain can be independently managed and evolved, for instance, with the introduction of new functionalities implemented in the context of the project.

Following the aforementioned approach, the testbed integrates a SUAV domain with a portable NFV infrastructure, enabling the creation of experimentation scenarios with mobile compute nodes. The portable NFV infrastructure encompasses six single-board computers, Raspberry Pi 3 Model B+, each with two Wi-Fi interfaces. Given their size and weight, these adequately represent the type of resource-constrained platforms that could be onboarded onto SUAVs. The infrastructure also includes five mini-ITX computers, which may serve as ground equipment to deploy more resource-demanding VNFs. To enable flight experiments, the portable NFV infrastructure is completed with four Parrot Bebop 2 SUAVs, each transporting a Raspberry Pi 3 Model B+. An additional mini-ITX computer hosts a VPN client and an OpenStack VIM, each running in a virtual machine. The former behaves as a network router with a virtual link to 5TONIC. The OpenStack VIM exposes the resources of the portable NFV infrastructure to the 5TONIC OSM stack.

On the other hand, the testbed includes the prototypes of a 3GPP UE and a 5G core network. The UE can also behave as an access router, supporting the connectivity of additional end-user devices. Both prototypes implement the data-plane protocol stack defined by 3GPP for an untrusted non-3GPP access [1]. The access router can be provisioned as a VNF at each network domain, in case an NFV infrastructure is available at the domain. Alternatively, it can be deployed as a hardware device, because an implementation of the access router has been made available on a single-board computer, Raspberry Pi model 3B+. Every access router function is connected to the 5G core network function through a GRE over IPsec tunnel, as dictated by 3GPP specifications for non-3GPP accesses. The 5G core network component has been provisioned as a VNF. Hence, different experiments can use independent instances of this network function, which can be deployed by the MANO platform on the 5TONIC NFV infrastructure. In addition, the 5G core network prototype implements a GRE/IPsec tunnel endpoint and provides connectivity to external networks. From that perspective, it can be seen as a baseline implementation of the data-plane protocol stack of a Non-3GPP InterWorking Function and a User Plane Function, as defined by 3GPP. The access router and the core network functions have been implemented using the Linux *ip-gre* module, and the *ipsec-tools* and *racoon* Linux packages. Additionally, a VNF has been implemented providing the functionalities of an IP telephony server based on the open-source SIP server *Kamailio*. It can be deployed over the 5TONIC NFV infrastructure, supporting the registration of end-user SIP phones and the establishment of voice and video calls in experimentation scenarios.

To enable experimentation activities with resource-constrained compute nodes (e.g., single-board computers onboarded on SUAVs), the testbed offers two additional VNFs: an Access Point VNF, providing the functions of a Wi-Fi access point and a DHCP server, and a Router/DNS VNF. Both VNFs have been prototyped as lightweight software functions using Linux and virtualization containers, so that they can be executed on the single-board computers of the SUAVs domain. All these components are available under an open-source license in the 5G-RANGE network layer repository [30].

## 3. Experimentation Scenario: Methodology from Design to Validation

**Figure 3:**
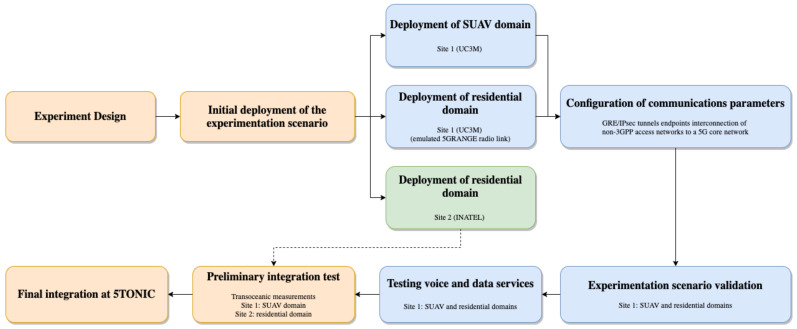
Methodology to define, deploy, integrate and validate the experimentation scenario.

Figure 1 shows the scenario that has been created with the goal of validating 5G-RANGE technologies in the context of a specific use case. The subsequent subsections detail the followed methodology from the design to validation. To facilitate the presentation of Section 3 and Section 4, and the understanding of the applied methodology, Figure 3 provides the overall flowchart illustrating the definition, deployment, integration and validation of the experimentation scenario.

### 3.1. Description of the Experimentation Scenario

**Figure 4:**
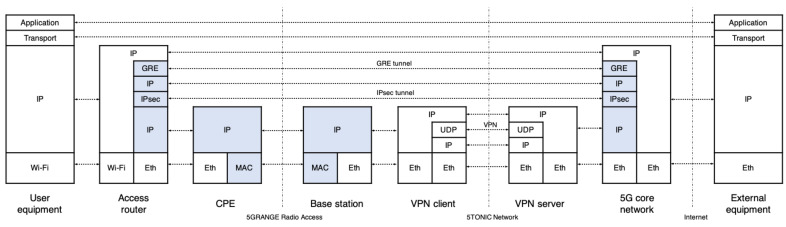
Data-plane protocol stack of the residential environment.

In the experimentation scenario, an access router is physically available at a residential environment in a remote area, serving as a wireless access point. The access router enables data exchange between residential users and external networks, through the GRE/IPsec tunnel established with the 5G core network. Hence, users within the remote area may access Internet and operator-specific services, such as web browsing, email, audio/video live streaming, or IP telephony. The radio access network is supported by a base station and a customer premises equipment (CPE), which implement the physical and MAC layer protocols of 5G-RANGE. Figure 4 shows the protocol stack involved at the different network functions of the residential domain, the access network, and the 5G core network to support data exchange.

The experimentation scenario also reproduces a situation where similar Internet and operator-specific services are to be provided to users beyond the limits of a 5G-RANGE radio cell (e.g., users in a festive event, or emergency response teams in a fire extinction or search and rescue operation). For this purpose, several SUAVs are deployed over the area, hosting a set of network functions that enable the provision of those services. In this case, the SUAVs are hovering in a static position, providing their intended services on a defined geographical area. These network functions include two wireless access points embedded as VNFs on two SUAVs, which serve as wireless hotspots to end users within their vicinity. A network router and a DNS server are jointly deployed as a VNF on a third SUAV. This VNF presents a virtual link towards an access router, which is virtualized on a ground equipment within the radio coverage of a 5G-RANGE radio cell (in a realistic scenario, this wireless link could rely on a multi-hop network path conformed by several wireless routers and SUAVs). The access router behaves as a GRE/IPsec tunnel endpoint towards the 5G core network, supporting data communications between end users and external networks. Communications among users in the residential and SUAV domains are supported through their radio access networks and the 5G core network.

### 3.2. Initial Deployment of the Experimentation Scenario

The experimentation scenario has been created as a composition of two network domains: one hosting the components of the residential environment (the residential domain); and a second one with the SUAV infrastructure (the SUAV domain). As a first step to build the experimentation scenario, we used our testbed resources to build the residential domain at 5TONIC. This domain includes two single-board computers (Raspberry Pi 3 Model B+), representing a 5G-RANGE base station and a CPE. This initial deployment of the experimentation scenario obviates the physical and MAC layer components of the 5G-RANGE access network (these will be integrated in the experimentation scenario at a later stage). Instead, the base station and the CPE are interconnected through a 100 Mb/s switch, providing the equivalent maximum throughput of the 5G-RANGE access network. The base station is connected to a mini-ITX computer, which deploys a VPN client. This implements a virtual link towards 5TONIC, making each device at the domain accessible from the laboratory. The CPE is connected to an access router function, supported by another single-board computer (Raspberry Pi 3 model B+). The access router provides the functions of a wireless access point, offering network access connectivity to an end-user device (a laptop), and implements a GRE/IPsec tunnel endpoint towards the 5G core network.

The 5G core network component is part of a network service that has automatically been deployed through the MANO platform of the 5TONIC NFV testbed. The network service includes a set of VNFs that are instantiated on the SUAV domain, offering the functionalities of wireless access points, routers, and other supporting functions on SUAV units. An access router VNF is deployed on a ground compute node (a mini-ITX computer), supporting the exchange of information with external networks. For this purpose, the access router behaves as a GRE/IPsec tunnel endpoint towards the 5G core network VNF. The latter is deployed at 5TONIC premises along with an IP telephony server VNF, which supports the establishment of calls among end users in our experimentation scenario. The deployment of the whole network service was accomplished at 5TONIC premises, where a specific location for indoor flights is available. Finally, an additional mini-ITX computer deploys the VPN client that handles the communication of the SUAV domain with other testbed components.

### 3.3. Configuration of GRE/IPsec Tunnel Endpoints

The use of GRE, IPsec, and the VPN service, may cause excessive fragmentation on data packets. When packets arrive to the access router or the 5G core network, they are fragmented by GRE before being processed by IPsec. This is because the default MTU of the GRE interface is 1476 bytes, a lower value than the typical size of regular data packets (1500 bytes). The GRE tunnel interface splits each data packet into two fragments, encapsulating them into new IP packets with a GRE and an outer IP header. With a size of 1500 bytes, the first of these packets is also required to be fragmented after being processed by IPsec (after appending the IPsec protocol overhead, the packet exceeds the MTU of the outgoing link). A similar situation occurs at the VPN endpoint, which processes three data packets and performs an additional fragmentation of the first packet (after appending the VPN protocol overhead, the first packet exceeds the MTU of the outgoing link). These fragmentation processes lead to increased overhead in terms of protocol headers and encryption of smaller packets, which necessarily impacts the achievable throughput.

As suggested in [31], this situation can be mitigated with an appropriate configuration of the MTU at the GRE tunnel interfaces. With a suitable value lower than 1500 bytes, data packets would only be fragmented at the GRE tunnel once, producing fragments with a size such that subsequent IPsec and VPN protocol overheads could be accommodated without additional fragmentation processes. Considering the reference MTU values indicated in [31], along with the protocol overhead of the VPN service, we have set the MTU on the GRE tunnel interface to 1360 bytes in our experimentation scenario.

For this purpose, we performed a set of experiments to verify their functional behavior and their performance in the provision of network communications. These experiments have also granted a better understanding on the impact of protocol overheads introduced by the GRE and IPsec processes needed to establish the proper connectivity towards the 5G core network, as well as of the VPN service that interconnects each network domain to the 5TONIC infrastructure. To evaluate the effect of this setting, we have done a performance evaluation, deploying all the elements of Figure 4 as virtual machines at the 5TONIC NFV infrastructure (except the CPE and the base station components). The deployment served to reproduce the communication scheme shown in the figure, including a virtual UE and a virtual external equipment, and excluding the components that are specific to the physical/MAC layers of 5G-RANGE (i.e., the physical/MAC and BS functions of Figure 4).

**Figure 5:**
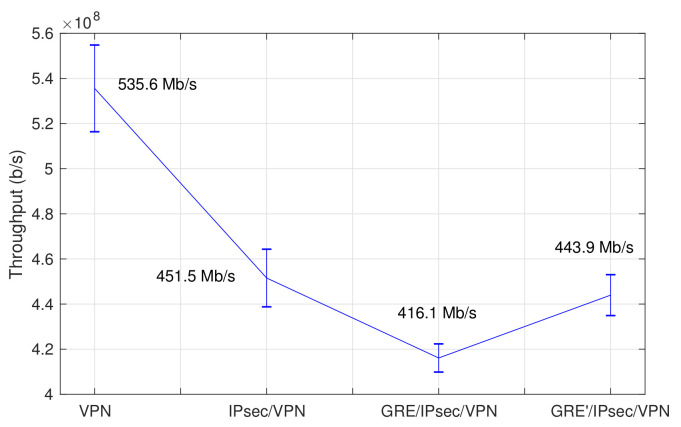
Performance evaluation of GRE/IPsec tunnel endpoints.

Figure 5 presents a synoptic overview of the results of our evaluation. Our tests with the *iPerf* tool show a maximum average throughput of 443.97 Mb/s between the user and the external equipment (*GRE’/IPsec/VPN* case in the figure), with an observable increase of approximately 6.7% with respect to the case where the MTU on the GRE tunnel interface is set to its default value of 1500 bytes (*GRE/IPsec/VPN*). These performance figures suggest the capacity of the access router and the 5G core network prototypes to accommodate the requirements for all the experimentation scenarios considered in 5G-RANGE, given that the maximum throughput required in a 5G-RANGE access network segment is 100 Mb/s. The optimized MTU value of 1360 bytes was used at the GRE tunnel interfaces in all our subsequent experiments. The errors bars correspond to the standard deviation.

To gain a better understanding of the performance overhead introduced by GRE/IPsec tunneling processes, the figure also presents the maximum average throughput for the cases where: GRE is disabled and the tunnel endpoints are only supported by IPsec (*IPsec/VPN* in Figure 5); and GRE/IPsec are disabled, and the access router and the 5G core network behave as network routers (*VPN* case).

### 3.4. Validation of the Experimentation Scenario

After the deployment of the experimentation scenario, we evaluated the throughput that could be achieved between a network domain and the 5G core network component. Our measurements with the *iPerf* tool reveal an available throughput of 248 Mb/s between a VPN client and the 5G core network VNF (these measurements are similar in both the SUAV and residential domains). This value is obviously lower than the already commented 443.97 Mb/s (see Figure 5) and this is because this new value corresponds to a measurement in a real network. In any case, it is still considerably above the data rate considered in the design of the 5G-RANGE radio access, i.e., 100 Mb/s.

As expected, the available throughput on the UAVs is lower than the available throughput in the VPN client as they communicate over a multi-hop ad hoc Wi-Fi network. The tests from the Access Point VNF SUAV result in average throughput of 22.7 Mbps, while the tests from the Router/DNS VNF SUAV have come out with 53.6 Mbps providing suitable values for subsequent experimentation activities.

On the other hand, tests with the Linux *Ping* command between the VPN client of the SUAV domain and the 5G core network VNF result in an average RTT of 1.59 ms, providing a low value that is suitable for subsequent experimentation activities. We want to highlight that in scenarios spanning remote domains, the network path between a domain and the 5G core network would be established across the Internet, being subject to network congestion and potential bandwidth limitations and high end-to-end network delays. Although this is not necessarily a limiting factor to build distributed experimentation scenarios, it should be considered when designing the tests that will be performed on top of them.

## 4. Multimedia Tests and Final Integration

### 4.1. Testing Voice and Data Services

Once the viability and suitable operation of both the access network and the extension of the network formed by the SUAVs have been confirmed, different multimedia services have been trialed. These tests have been also replicated with the real radio equipment, once it was integrated in the laboratory for a project review demonstration. The experimentation scenario has been used to test a representative set of voice and data services that might be demanded by end users in remote areas. This has served to verify the appropriateness of our testbed to develop proof-of-concept activities in the context of a specific use case.

**Figure 6:**
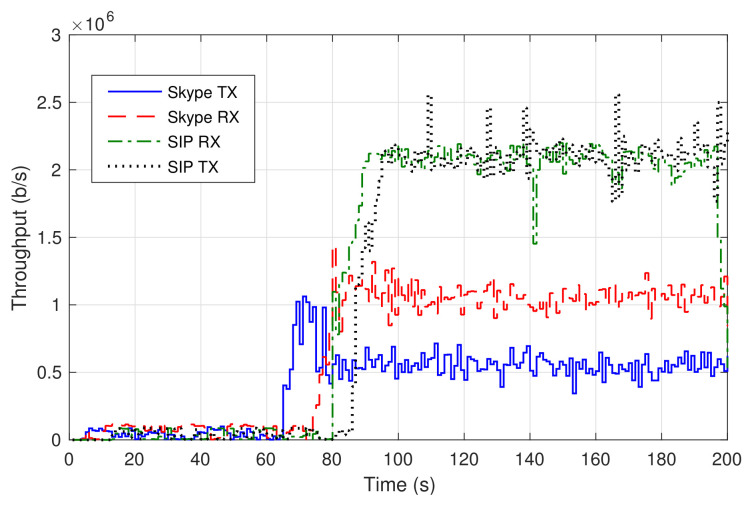
Data rates of SIP and Skype calls.

First, we established a voice call between a user in the residential domain and a user in the SUAV domain using a softphone based on the SIP protocol (Session Initiation Protocol) [32], *Bria*, which was installed on one laptop in each domain. The softphones were configured to use the IP telephony service VNF of Figure 1. Figure 6 represents the transmission rate (*SIP TX*) and the received throughput (*SIP RX*) of the voice traffic, measured at the laptop in the residential domain. Around second 75, the video was turned on in both softphones, resulting in a traffic increment (from a few Kb/s up to approximately 2 Mb/s). The average *jitter* in both directions was 0.5 ms, resulting in appropriate interactive real-time communications. The user experience during the call has been satisfactory, with no audio glitches nor skipped video frames.

In a second experiment, a group call was set up using Skype. The call involved the same users as in the previous experiment, with an additional third user connected to Skype through an external network (a commercial fixed access). Figure 6 shows the transmission rate and the received throughput of the voice traffic observed by the residential domain user. The video was activated approximately within 70 s, causing a consequent increase of the traffic. The received traffic nearly doubles the transmitted traffic because, in a Skype video conference, the video stream of each participant is routed to an external server cluster, which in turns forwards it to every other participant. That way, the received throughput corresponds to the video transmitted by the other two participants in the call.

**Figure 7:**
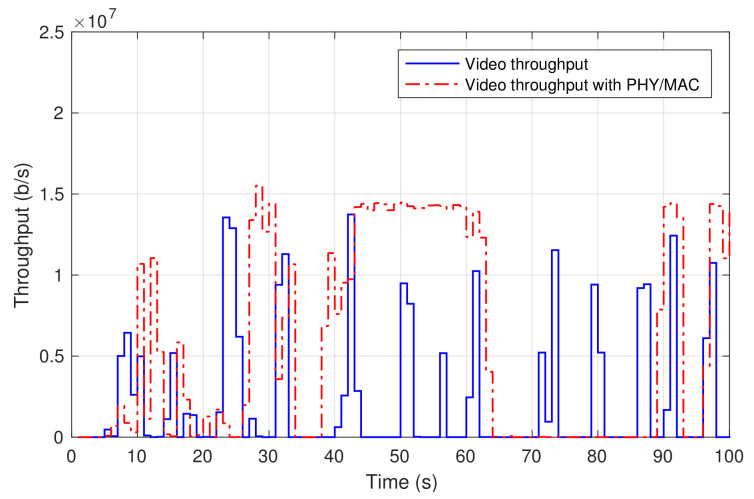
Data rates of video-on-demand service.

Finally, the laptop at the residential domain was used to access a 4 K video from YouTube. Figure 7 shows the throughput of the received video at the laptop (labelled as *video throughput*). The video was continuously played out with no freezing nor skipped video frames.

### 4.2. Final Integration and Validation

In the previous section, we verified the appropriateness of our testbed to develop proof-of-concept activities in the context of a specific use case. In this section, we present the methodology followed to integrate the prototypes of a 5G-RANGE base station and a CPE into the experimentation scenario. These prototypes are available at Inatel in Brazil, and were temporarily brought to 5TONIC for a demonstration.

The transceiver prototype [15] has been developed using the software-defined radio strategy, where the entire base band processing is implemented using C language over GNU Radio platform. This approach leads to the maximum flexibility, since the radio behavior can easily be adapted to the different channel conditions. In this prototype, data from the network layer is delivered to the MAC layer through an Ethernet connection. The MAC layer shares the physical resources for the different users according to their individual throughput demands and channel responses. Adaptive modulation and coding is used to guarantee a desired quality-of-service in terms of bit error rate. Modulation order and coding rate for each user is automatically defined based on the channel quality report provided by the mobile terminals. Once the data is mapped to the physical resources, the information is processed by the channel encoder and modulated using generalized frequency division multiplexing, an innovative waveform capable of providing robustness against doubly dispersive channels. In this prototype, a 2 × 2 MIMO system has been implemented, providing spatial multiplexing gain for users that are nearby the base station and diversity gain for those located far away from it. Once the data is processed by the base band unit, it is delivered to the digital-analog converter, coupled with the radio frequency head. In our implementation, universal software radio peripheral are used to receive the digital samples from the computer running the transceiver’s MAC and physical cores, converting them to the radio frequency signal to be delivered to the transmit antennas. A power amplifier provides 6 watts per antenna, allowing the signal to be received up to 50 km from the base station, with data rates up to 100 Mb/s over a 24 MHz channel. Spectrum sensing is also implemented using the software-defined radio approach. It is performed by the mobile terminals and the collected measurements are periodically sent to the base station using the control channel. The MAC layer uses this information to decide which channels are available to be allocated to the users.

To facilitate integration activities, we leveraged the capacity of our NFV testbed to incorporate remote network domains. In particular, the residential domain of Figure 1 was initially replicated at Inatel, replacing the single-board, back-to-back connected computers by the base station and the customer premises equipment prototypes. The VPN client at Inatel was configured with the same security credentials as the VPN client of the 5TONIC residential domain. All the equipment and networks at the Inatel domain were configured with the same IP addresses as their correspondent equipment and networks at the 5TONIC residential domain. An access router function was also deployed. To ease the deployment of this function, the memory card of the device providing the access router at 5TONIC was cloned, being installed on a Raspberry Pi 3 model B+ at Inatel.

**Figure 8:**
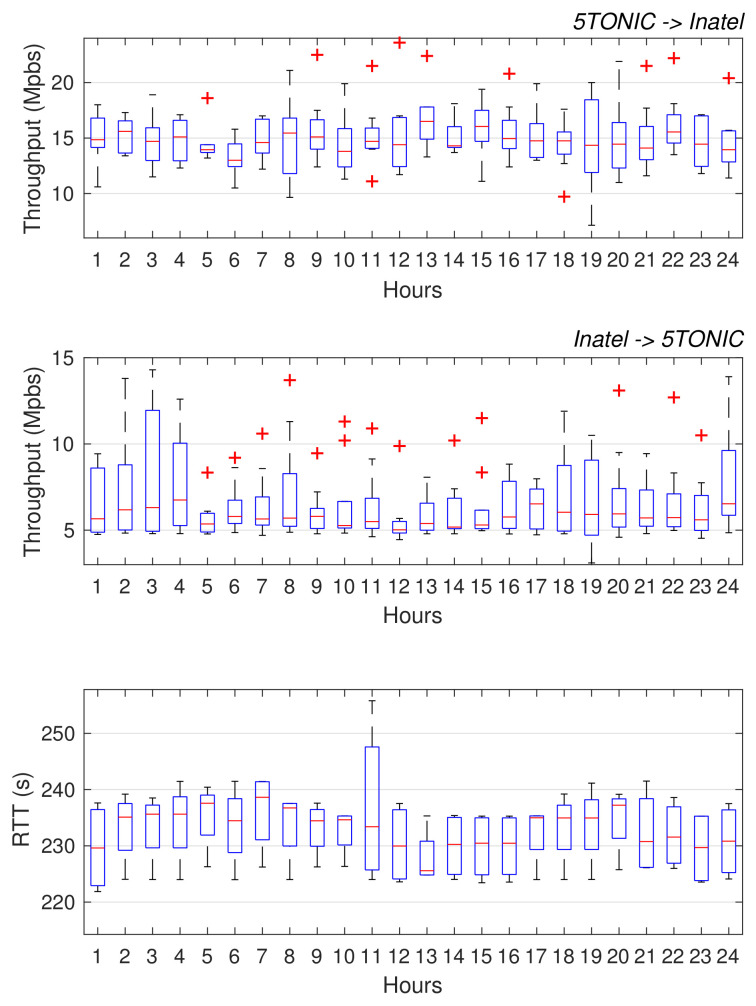
Transoceanic network path performance between 5TONIC and Inatel.

We want to highlight that this methodology allowed addressing most of the integration aspects in advance, before the physical/MAC layer prototypes were brought to 5TONIC. Following this method, we have performed a straightforward final integration. Otherwise, following a traditional methodology, several configurations must be made after the integration into a single local demonstrator, such as (i) configure the addressing space, (ii) configure the GRE/IPsec tunnel, (iii) the integration of physical, MAC, and network layer components, or (iv) the overall validation with voices and data services. This work would be even more time-consuming in this case since the institutions are located on different continents. Thanks to the proposed methodology, all these assignments have been configured prior to the final integration. In addition, it enabled the realization of preliminary configuration and tests, verifying the proper interaction among the components at the Inatel residential domain and those available at 5TONIC. Taking advantage of the VPN service, these tests were conducted as if the Inatel residential environment was locally available at 5TONIC. Of course, the aforementioned tests were limited by the performance of the transoceanic network path between 5TONIC and Inatel, which supports an average throughput of 15.07 Mb/s in the 5TONIC to Brazil direction, 6.62 Mb/s in the reverse direction, and an average RTT of 232.46 ms (these values were obtained during a period of 20 days, taking measurements every hour). This information can be appreciated in Figure 8. The data is plotted following the standard boxplot shape, which represents the obtained measurements of the available throughput grouped in quartiles.

When the physical/MAC layer equipment was brought to 5TONIC, their integration into the experimental scenario only required the decommission of the two single-board, back-to-back connected computers of the 5TONIC residential environment, which were simply replaced by the physical/MAC layer prototypes. The whole process could be realized in a reduced time frame (less than half working day), making the whole experimentation scenario rapidly ready for the practical demonstration. As an example, Figure 7 also shows the throughput of a 4K video received from YouTube at the 5TONIC residential domain. The video was delivered through the base station and the customer premises equipment prototypes, being uninterruptedly played out and with an appropriate user experience. The traffic pattern is similar to the one shown in the previous section, although not identical, since both experiments were conducted at distant moments in time, using different videos.

## 5. Conclusions and Future Work

This article presents an experience report after building a use case practical scenario to trial 5G network technologies for remote areas. The scenario was created using an existing NFV testbed that supports the flexible incorporation of network domains, this way easing integrating activities. Our experience suggests that the use of distributed network domains has the potential to reduce implementation and test cycles, providing realistic and moderately complex scenarios to stakeholders, who may test new developments along with other assets available at the different domains. In addition, it facilitates joint demonstration activities, as any remote domain can easily be redeployed at a central location (i.e., 5TONIC in case of the 5G-RANGE project) using the same security credentials. The design and development of the different parts of the experiment have been done remotely and later integrated together. This is possible thanks to the use of a VPN established from each site towards the 5TONIC laboratory, which holds the 5Gcore functionality, allowing the integration of the different network domains. This procedure facilitates the preliminary validation analyses to enable a fast and straightforward real integration procedure. Also, in this article, we have proved with commodity equipment that both (i) to comply with 3GPP/5G standards, and (ii) to use the VPN as an integration and deployment tool, does not limit the system performance.

Our future work will explore the potential of the 5G-RANGE testbed to develop experimentation scenarios for other innovative use cases in remote areas. In addition, we will work on the evolution of the testbed, considering new releases of its software base (i.e., OSM and OpenStack), as well as emergent open-source virtualization technologies for resource-constrained mobile nodes (e.g., Kubernetes and fog05).

## Data Availability

Not applicable.

## Abbreviations

The following abbreviations are used in this manuscript:

5G	Fifth generation of cellular network technology
3GPP	3rd Generation Partnership Project
eMMBB	enhanced Mobile Broadband
URLLC	Ultra-Reliable Low Latency Communications
mMTC	massive Machine Type Communications
MAC	Medium Access Control
5TONIC	5G Telefonica Open Network Innovation Centre laboratory
SUAV	Small Unmanned Aerial Vehicle
QAM	Quadrature Amplitude Modulation
MIMO	Multiple-Input Multiple-Output
NFV	Network Functions Virtualization
VNF	Virtual Network Function
MANO	Management and Orchestration
OSM	Open-Source MANO
VPN	Virtual Private Network
CPE	Customer Premises Equipment
SIP	Session Initiation Protocol
IPsec	Internet Protocol security
GRE	Generic Routing Encapsulation
